# 2830. Clinical Characteristics, Outcomes and Antimicrobial Resistance of Non-aeruginosa Pseudomonas Infection in Adult Cancer Patients

**DOI:** 10.1093/ofid/ofad500.2440

**Published:** 2023-11-27

**Authors:** Hyundam Gu, Sadaf Aslam, Cody Horn, John Greene

**Affiliations:** Academic Visitor & Scholar Program, Moffitt Cancer Center; University of South Florida Morsani College of Medicine, Tampa, Florida; Adena Regional Health System, Chillicothe, Ohio; Moffitt Cancer Center, Tampa, FL

## Abstract

**Background:**

Pseudomonas aeruginosa a gram-negative bacteria that is known to cause severe nosocomial infections. Other species of Pseudomonas are considered to be part of the human indigenous microbiota, commonly found in sites such as the gastrointestinal tract and skin. Although believed to be non-pathogenic for many years, these species can cause diseases in immunocompromised patients, albeit less virulent than P. aeruginosa. In this report, we will discuss the characteristics of non-aeruginosa Pseudomonas infections in cancer patients.

**Methods:**

We conducted a retrospective study investigating culture results of adult inpatients with Pseudomonas spp. infections, excluding P. aeruginosa, at a Florida cancer center from 2012-2022. Data collected included infection source, antibiotics used, resistance against fluoroquinolones, surgical procedures or chemotherapy in the past month, polymicrobial infection, and 30-day mortality.

**Results:**

Of 104 cases, P. putida was the most common species, followed by P. fluorescens, P. stutzeri, and P. mendocina. P. putida infections were often associated with urine (44%), respiratory and skin wounds (16% each). Respiratory tract was the most common source for P. fluorescens infections (38%). Solid tumors accounted for 71% of malignancies in the P. putida group, while hematologic malignancies accounted for 29%. For P. fluorescens, solid tumors and hematologic malignancies accounted for 62% and 38%, respectively. P. stutzeri and P. mendocina were mostly associated with solid malignancies. All groups were susceptible to quinolones (86-100%), and polymicrobial infections were frequently observed (68%, 73%, 63%, and 50%, respectively). P. putida and P. fluorescens had 7% and 19% 30-day mortality rates, respectively. P. stutzeri and P. mendocina had no observed mortality.

**Figure:**
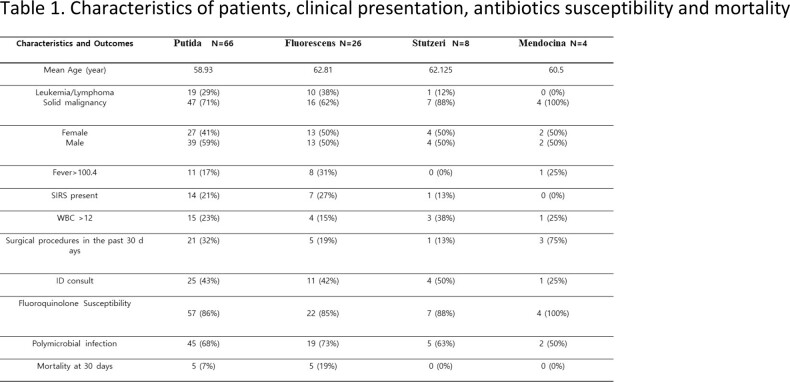

**Figure:**
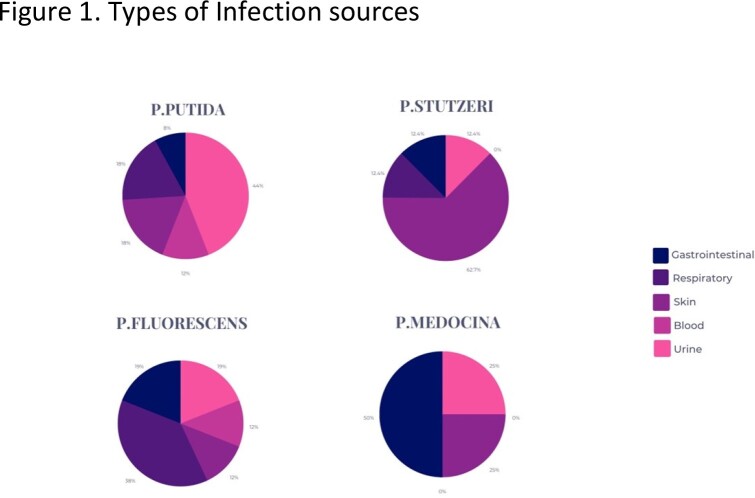

**Figure:**
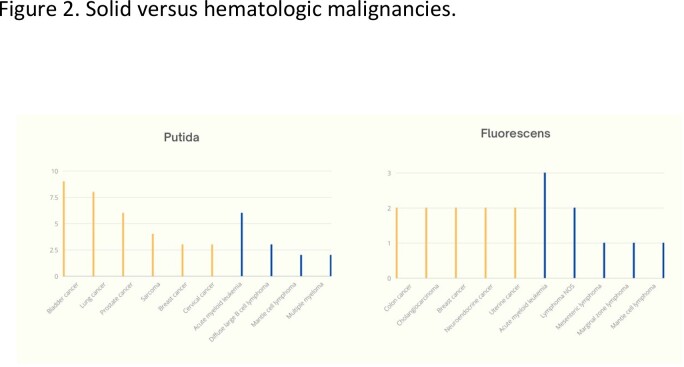

**Conclusion:**

Our study highlights non-aeruginosa Pseudomonas infections are rare but notable in cancer patients. In the majority of cases, the disease course is less severe, and treatment with appropriate antibiotics, such as fluoroquinolones, leads to complete recovery.

**Disclosures:**

**All Authors**: No reported disclosures

